# Mitochondria: The Crossroads of Complement Activation and Kidney Injury Progression

**DOI:** 10.3390/ijms27125599

**Published:** 2026-06-21

**Authors:** Madison K. McGraw, Nirmala Parajuli

**Affiliations:** Department of Pharmacology and Toxicology, University of Arkansas for Medical Science, Little Rock, AR 72205, USA; mkmcgraw@uams.edu

**Keywords:** kidney pathology, complement system, mitochondria, acute kidney injury, chronic kidney disease, C5, C5aR1

## Abstract

Acute kidney injury, a broad term associated with diverse etiologies, is a common pathological condition that develops into chronic disease via mechanisms that have yet to be fully understood. Key processes that promote chronic disease transition include mitochondrial dysfunction and aberrant complement system activation, specifically inducing inflammation and accumulation of pro-fibrotic changes. Although emerging evidence strongly indicates that these two processes are closely intertwined, identification of appropriate therapeutic targets remains limited. Among complement proteins, terminal portions of the cascade, including complement 5 (C5), exert particularly robust effects on mitochondrial function across tissues, including the kidney. Moreover, C5 is the most terminal portion of the cascade to produce a highly pro-inflammatory anaphylatoxin, positioning C5 as an ideal clinical target during kidney injury/disease. In this review, we will hence summarize current knowledge regarding mitochondrial contributions to kidney pathophysiology through the lens of the close relationship between mitochondria and the complement system, particularly C5.

## 1. Introduction

The development of kidney disease from acute injury is a cost-intensive and deadly progression associated with high patient mortality [1,2]. Despite this, there are few treatment options available that address disease development and preserve life. Currently, clinicians rely upon renal replacement therapies such as dialysis [3] or kidney transplantation [4], which only come into play after kidney disease has reached an advanced stage. There is, therefore, an emerging area of research that seeks to identify mechanisms and biomarkers associated with the transition from acute kidney injury to chronic damage/disease. Prior studies identify that metabolism, particularly mitochondrial function, is closely intertwined with both acute and chronic renal injury [5,6,7]. Dysfunctional mitochondria mediate direct damage to cells, leading to the release of pro-inflammatory factors, which interface with the immune system. Aberrant activation of immune system components promotes excessive scar formation, causing a permanent loss of renal functional capacity, which predisposes the tissue to further injury [8,9,10]. One of the main immune effectors governing this change is the complement cascade, which itself contains pro-inflammatory signaling molecules [10,11,12,13]. However, ongoing work suggests that complement proteins perform non-canonical functions intracellularly, including regulation of critical physiological processes such as metabolism [14,15,16].

In this review, we discuss the central role of mitochondria in kidney disease development and examine current evidence of complement’s involvement in mitochondrial pathophysiology. We furthermore focus on the known contributions of the terminal complement cascade protein C5 to kidney injury, including mitochondrial dysfunction, as an optimal therapeutic target.

## 2. Renal Function and Disease Transition

The kidneys are critical organs for overall health with diverse functions to maintain homeostasis, including the removal of waste, acid/base balance, blood pressure control, electrolyte regulation, and much more [17]. Therefore, it is no surprise that kidney disease is currently the 9th leading cause of death in the United States and is predicted to become the 5th leading cause by 2040 [18]. Patients with end-stage disease require kidney replacement therapies such as dialysis; however, these interventions are associated with substantial healthcare costs, reduced quality of life, and high mortality rates [4,19]. While kidney transplantation remains the optimal treatment, limited organ availability underscores the urgent need for strategies that prevent disease progression.

Kidney disease frequently begins with a single acute insult, most commonly acute kidney injury (AKI). In many cases, maladaptive repair following AKI—characterized by persistent inflammation, fibrosis, and incomplete tissue regeneration—leads to long-term functional impairment and markedly increases susceptibility to subsequent AKI episodes. This cycle accelerates progression to chronic kidney disease (CKD) and ultimately end-stage kidney disease (ESKD). Despite extensive efforts to identify biomarkers of AKI, effective preventative or disease-modifying therapies remain lacking. Thus, to delay the progression to ESKD in patients, it is critical to identify and target mechanisms that drive the transition from AKI to sustained CKD.

### 2.1. End-Stage Kidney Disease: Epidemiology and Etiologies

ESKD, also referred to as end-stage renal disease (ESRD), represents the terminal stage of a progressive kidney dysfunction arising from diverse etiologies. Defined by an estimated glomerular filtration rate (eGFR) [18] of less than 15 mL/min/1.73 m^2^, ESKD patients lack adequate blood filtration capacity, allowing the buildup of uremic toxicants such as low-molecular-weight solutes, protein-bound solutes, and middle molecules (>500 kDa). Over 100 unique uremic toxicants have been identified, and if left untreated, the accumulation of these toxicants is life-threatening [20].

Treatment modalities for ESKD are limited to dialysis or transplantation. Although dialysis provides life-sustaining filtration, it is a resource-intensive, temporary solution and cost-intensive therapy that does not successfully remove large uremic toxicants [20,21]. Kidney transplantation offers superior long-term survival; however, organ shortages, strict qualification guidelines, and other complicating factors limit access for many patients [3,22,23]. Currently, over 800,000 people in the United States are diagnosed with ESKD [1,24] and ESKD ranks as the 9th leading cause of death among all diseases. In response to the rising burden, the Executive Order on Advancing American Kidney Health was enacted in 2019 with the goal of reducing the ESKD incidence by 25% by 2030 [25] and expanding access to home dialysis and transplantation. While organ donation rates have improved by >30% in recent years, the overall incidence of advanced kidney disease continues to increase rapidly, and is estimated to continue to significantly impact public health [26].

**Figure 1:**
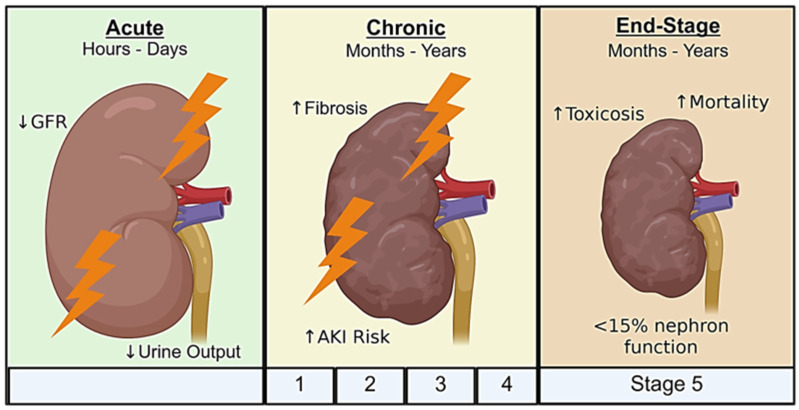
Schematic of ESKD progression. After acute kidney injury (AKI) damages the tissue, improper wound healing can develop into a chronic scar (CKD) that affects kidney function and increases the risk for subsequent AKI’s. Stages 1–4 of CKD are determined by the eGFR and albuminuria. Stage 5 kidney disease is referred to as “end-stage” (ESKD) and represents a point of nephron function loss that requires renal replacement therapy to sustain life. Created in BioRender. McGraw, M. (2026) https://BioRender.com/ygn1way.

The etiologies for ESKD can be varied, encompassing various comorbid conditions or insults, including diabetes, hypertension, infection, or drugs/toxins [18]. Progression to ESKD is typically preceded by CKD, which itself is commonly initiated or accelerated by AKI (Figure 1).

#### 2.1.1. Acute Kidney Injury

AKI is the most critical independent risk factor for progression to CKD. Thought to affect more than 13 million people worldwide each year, AKI is characterized by a rapid decline in kidney function and is clinically defined by an increase in serum creatinine (SCr; more than 0.3 mg/dL increase in 48 h) and/or decreased urine output [27]. AKI is most commonly recognized in hospitalized patients, with an incidence of up to 20% among all hospitalized individuals and as high as 67% among intensive care unit patients [28]. The condition arises from varied etiologies that contribute to its complex pathogenesis.

AKI is broadly categorized as prerenal, intrarenal (intrinsic), or postrenal. In practice, these categories share overlapping pathophysiological features, and two or more mechanisms may contribute simultaneously to AKI development. As such, differential diagnoses must account for complex and often multifactorial etiologies. Among hospitalized patients, prerenal causes account for up to 60% of AKI cases, making this category the largest contributor overall [29]. Prerenal AKI results from inadequate perfusion, leading to tissue injury. Common causes include blood volume depletion due to dehydration or hemorrhage [30,31], cardiorenal syndromes such as congestive heart failure [32], and decreased vascular resistance from sepsis [33,34]. In many cases, the primary source of renal injury is not the poor perfusion itself, but rather the inflammatory and necrotic damage that occurs after reperfusion. This phenomenon is well characterized as ischemia and reperfusion injury (IRI) [35].

In contrast, intrarenal AKI is defined by injury to specific components of the kidney, including the glomeruli, tubules, or vasculature, and is often secondary to another insult. Acute tubular necrosis (ATN) is the most common feature (>80%) [36,37]. Postrenal AKI results from extrarenal obstruction, most commonly due to blockage along the ureter or urethra, and is typically differentiated easily from the other AKI categories. Thus, we focus on the AKI subtype IRI, a prerenal insult that subsequently drives intrarenal ATN.

#### 2.1.2. Ischemia-Reperfusion Injury

The two phases of IRI (ischemia and reperfusion) involve different pathological mechanisms that cumulatively exacerbate renal cell death and tissue injury. During the ischemic phase, a mismatch between oxygen supply and demand causes metabolite accumulation and ATP depletion. Depending on the length of time the oxygen supply is compromised, widespread necrosis (i.e., ATN) can occur [38]. Signals during the ischemic phase can also drive the activation of other cell death programs, among which apoptosis is the most common [39]. However, further studies have implicated a variety of regulated cell death mechanisms that contribute to IRI, including pyroptosis [40], necroptosis, and ferroptosis [41]. While these “regulated necrosis” mechanisms play a significant role in responses to pathogenic infection, as discussed by Zhang et al. [42], they all share common morphologic features with necrosis, including cell membrane rupture [43]. Notably, inhibition of regulated necrosis mechanisms such as ferroptosis demonstrates protective effects even during severe models of renal IRI [44]. The release of cytosolic contents into the extracellular space is a potent stimulator of inflammation [45,46], particularly when blood flow to the kidney is restored.

During the reperfusion phase of IRI, released cell contents such as fragmented DNA, mitochondrial content, and ATP (referred to as damage-associated molecular patterns, or DAMPs) are recognized by immune cells from the newly restored blood supply [47]. For example, many DAMPs have been shown to interact with pattern-recognition receptors (PRRs), initial sensors for infection that coordinate immune inflammatory responses [48]. “Sterile” inflammation ensues, and its resolution (or lack thereof) is a major determinant of IRI severity as well as the risk for long-term complications.

#### 2.1.3. Chronic Kidney Disease

While AKI (and therefore, IRI) represents a sudden and often reversible loss of kidney function that spans between hours and days, CKD is characterized by a gradual and irreversible decline in kidney function. While the two pathologies share similar symptoms, a diagnosis of CKD is usually a function of time—for example, an eGFR of <60 mL/min/1.73 m^2^ for longer than 3 months [49]. Thus, CKD progression is stratified into stages 1 through 5, with the fifth (final) stage corresponding to ESKD [50,51].

A prior episode of AKI, regardless of etiology, is the primary risk factor for the subsequent development of CKD. Although the mechanisms linking IRI to long-term decline in renal function remain incompletely understood, the severity of the injury is strongly correlated with the risk of CKD progression [52]. Furthermore, established CKD increases the risk of recurrent AKI by up to tenfold, creating a vicious cycle of injury that culminates in fibrosis and gradual loss of renal function [53]. These observations underscore the urgent need to elucidate factors influencing IRI severity, as such insights could reduce IRI-associated mortality and slow the progression of CKD.

#### 2.1.4. Gaps in Knowledge

One of the major challenges in developing therapeutic strategies for kidney disease is that the determinants of disease progression remain incompletely understood. For example, it is unclear why AKI (and IRI) results in fibrosis and maladaptive repair in some patients, resulting in long-term functional decline, while others experience full recovery [9,54]. Many of the biological modifiers implicated in these fibrotic changes are pro-inflammatory, including mitochondria-derived oxidative stress [5] and the overactivation of innate immune pathways, such as the complement cascade [10]. However, rather than broadly and non-specifically blunting these critical systems, a more targeted therapeutic approach to the transition from AKI to CKD is essential. Identifying modifiable pathways may enable precise interventions that reduce fibrosis, preserve nephron mass, and ultimately prevent progression to ESKD.

## 3. Renal Mitochondria

Mitochondria are specialized organelles that are primarily responsible for converting nutrients (glucose, fatty acids, etc.) into the main cellular energy currency, adenosine triphosphate (ATP). This process occurs predominantly through aerobic oxidative phosphorylation (OXPHOS), making mitochondria central to cellular energy homeostasis in the kidney.

### 3.1. Electron Transport and Oxidative Phosphorylation

**Figure 2:**
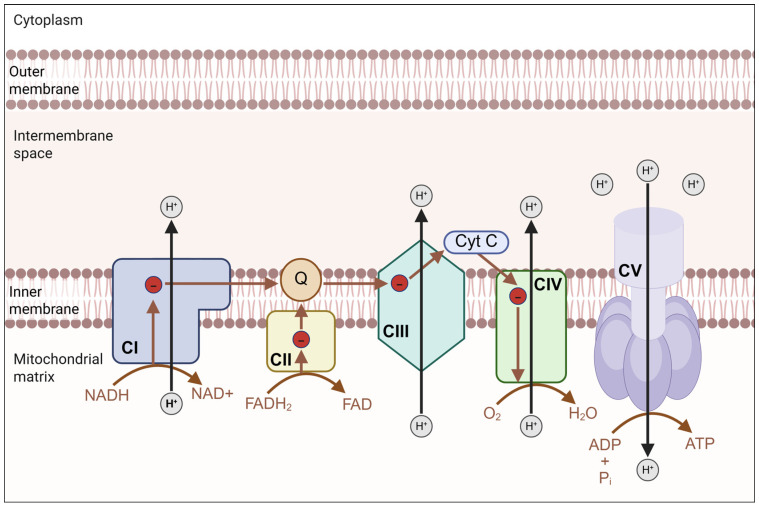
Schematic of oxidative phosphorylation (OXPHOS). Created in BioRender. Mcgraw, M. (2026) https://BioRender.com/sb609uu.

The mechanism of OXPHOS (Figure 2) has been extensively characterized. First, electrons derived from oxidizable substrates are transferred through a series of protein complexes embedded within the inner mitochondrial membrane, collectively termed the electron transport chain (ETC). This favors the pumping of protons into the inner membrane space by ETC complexes I, III, and IV, generating a charge difference between the inner membrane space and the mitochondrial matrix called the proton gradient. Protons then flow back into the matrix through complex V (ATP synthase), and this proton-motive force drives the synthesis of ATP from adenosine diphosphate (ADP) and inorganic phosphate (P_i_).

### 3.2. Regulation of Oxidative Phosphorylation

The proton gradient, also referred to as the proton motive force, is an essential component of OXPHOS that enables the production of approximately 90% of the ATP in the body [55]. It is made up of two parts: (1) a mitochondrial membrane potential, which is the primary contributor to the proton gradient, and (2) a pH gradient across the inner mitochondrial membrane [56]. Proton pumping by the ETC, as discussed, is the foremost contributor to the mitochondrial membrane potential and therefore, the proton gradient. However, other mechanisms can and do play a role in mediating the proton gradient, including ATP synthesis (gradient usage) and uncoupling (gradient dissipation) [57,58]. Thus, it is helpful to examine OXPHOS through a lens of proton gradient regulation.

#### 3.2.1. Mitochondrial Complexes and Supercomplexes

For many years, ETC complexes were thought to exist as discrete, free-floating entities within the inner membrane (termed the “fluid state” model of random diffusion) [59]. However, this view was later challenged when evidence of higher-order organization of ETC complexes emerged, and the “solid state” model was proposed [60]. This model purported that ETC complexes were organized into stable multimeric structures, so-called “supercomplexes,” which brought complexes into physical proximity to enhance the efficiency of electron transport [60]. Current understanding favors a “plasticity” model, wherein ETC complexes dynamically exist either as individual units or as supercomplexes depending on metabolic demands [61]. IRI is a known contributor to supercomplex destruction [62]. However, relatively few studies address the role of supercomplexes during IRI, including impacts on the proton gradient and OXPHOS.

#### 3.2.2. The Proton Gradient and IF-1

In addition to proton pumping by the ETC, other major mechanisms regulating the proton gradient (and thereby, OXPHOS) include uncoupling and ATP synthesis. Mitochondrial “uncoupling,” carried out by uncoupling proteins (UCPs), is defined as leakage of protons back into the mitochondrial matrix and the release of the resulting energy as heat [63]. Rapid uncoupling results in robust ATP depletion and, potentially, cell death [63]. However, other reports suggest that controlled uncoupling contributes to important physiological processes [63,64,65].

Other than dissipation of the proton gradient via uncoupling, protons can also travel back to the mitochondrial matrix through the ATP synthesis process. The ATP synthase, which performs this key function, is made up of two functional units: the F_1_ catalytic component, which is situated in the matrix, and the F_0_ sector bound within the inner mitochondrial membrane [66,67]. Sufficient proton-motive force causes protons to pass through F_0_, providing the energy required for the rotation of motor machinery. The rotation of F_1_ subunits then catalyzes the ATP synthesis reaction [58,68]. Additionally, the hydrolysis of ATP into ADP and P_i_ at the F_1_ catalytic site fuels a reverse mechanism whereby protons can be pumped by the enzyme from the matrix into the intermembrane space [69]. Thus, ATP synthase, in conjunction with the factors that regulate it, forms another important arm through which the proton gradient can be modified.

**Figure 3:**
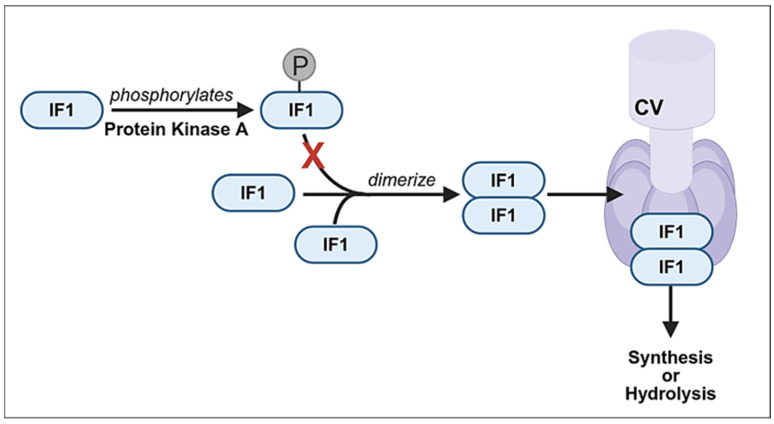
Schematic of IF1 dimerization and interaction with complex V. Created in BioRender. McGraw, M. (2026) https://BioRender.com/4ea99w8.

Beyond the ATP synthase itself, one of the most well-known factors contributing to ATP production is ATPase Inhibitory Factor 1 (IF1). Originally discovered as an inhibitor of ATP hydrolysis, this small ~16 kDa protein has since been investigated as a modulator of both ATP synthesis and hydrolysis [70,71]. Canonically, IF1 forms an active dimer in acidic conditions and binds to a site on the catalytic head of complex V (Figure 3), sometimes interacting with multiple complex V catalytic subunits at the same time [72]. Other conditions, such as dephosphorylation of IF1 at the S39 residue, must be met before this interaction can occur [73].

**Figure 4:**
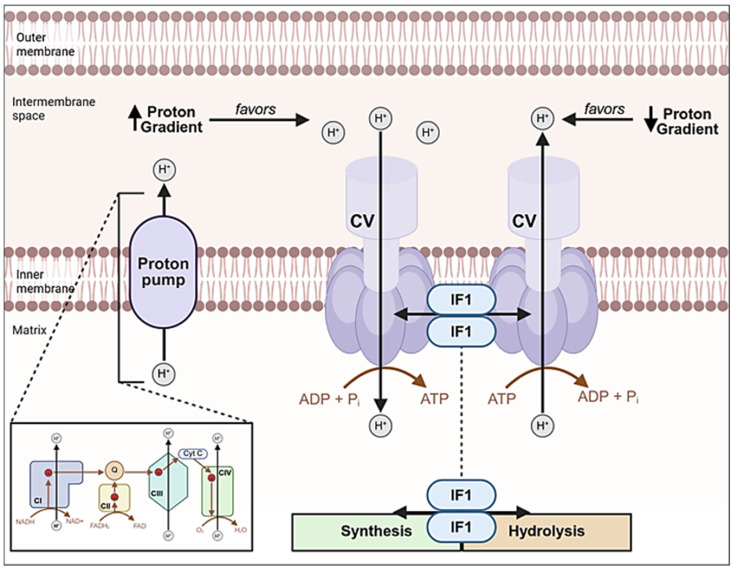
Generation of the proton gradient and regulation by IF1. Created in BioRender. McGraw, M. (2026) https://BioRender.com/lflsnzg.

Once IF1 interacts with complex V, it acts as a master regulator of ATP synthesis and hydrolysis. As a consequence, IF1 also influences the mitochondrial membrane potential (Figure 4), as facilitating ATP synthesis results in proton movement into the mitochondrial matrix while promoting hydrolysis pumps protons back into the intermembrane space [74]. Beyond IF1’s canonical role as a factor of complex V, upregulation of the IF1 protein has also been associated with increased glycolysis in prior studies [70,75,76]. This includes aerobic glycolysis, otherwise known as the ‘Warburg effect’ [70,76]. Discovered in 1924 by Otto Warburg, this phenotype was first defined in cancer cells and tumors, which preferentially utilize glycolysis for the production of ATP, even when sufficient oxygen is present [77]. Ultimately, this shift in metabolism increases cell survival and proliferation. This is because, although aerobic glycolysis is an inefficient means of generating ATP per unit of glucose, the rate of glucose metabolism via aerobic glycolysis is 10–100 times faster than OXPHOS [78]. Together, these observations position IF1 as a central regulator of mitochondrial energetics capable of coordinating ATP synthesis, hydrolysis, and metabolic reprogramming under stress conditions.

#### 3.2.3. The Complex Role of Mitochondrial ROS

“Oxidative stress” within cells is attributed to the excessive formation of reactive oxygen species (ROS). The mitochondria are the predominant source of endogenous ROS, which is produced as a normal consequence of OXPHOS. Electrons leaking from the ETC are accepted by oxygen, generating a superoxide anion (O_2_^•−^). In healthy cells, O_2_^•−^ is routinely scavenged by superoxide dismutase enzymes (SODs) and is eventually converted to H_2_O [79,80]. However, unscavenged superoxide can readily react with nitric oxide, forming the extremely damaging species peroxynitrite (ONOO^−^) [81]. Owing to the deleterious impact of excessive O_2_^•−^ production, mice deficient in SODs demonstrate significant cardiovascular injury, neural degeneration, and lethality [82,83]. Similarly, oxidative stress is closely linked with renal IRI pathology [35,84].

While ROS was originally characterized for its damaging effects, it is important to note that regulated ROS production plays several key roles within healthy cells. Intracellular ROS contributes to redox signaling pathways, affecting a variety of biological activities [85]. ROS levels determine the oxidation of key phosphatases and kinases, regulating enzyme function [86,87]. Cellular redox status can also influence transcription factor translocation to the nucleus [88] and mediate certain epigenetic modifications [89,90]. Thus, ROS production is a critical component for broad cellular signaling, governing cell stress responses [91,92], inflammation [93,94], metabolism [88], and homeostasis [95]. Imbalances in ROS production, such as during IRI, carry consequences for a wide range of pathways in addition to direct cellular damage.

### 3.3. Mitochondrial Dysfunction in Acute Kidney Injury and Chronic Kidney Disease

As an integral part of cellular metabolism, mitochondrial dysfunction is a common feature of many pathologies and is particularly pronounced in high-energy demand organs such as the kidney [6]. In addition, mitochondrial damage and loss of function are known factors contributing to AKI, as well as the transition to chronic disease [5].

#### 3.3.1. Mitochondrial Damage During IRI

Mitochondrial damage during IRI suppresses OXPHOS, leading to rapid ATP depletion [6]. This disruption also triggers robust mitochondrial ROS generation, damaging ETC complexes—especially complexes I and III, which are major sites of ROS production [96,97]. This injury is accompanied by loss of cristae architecture and the depolarization of the mitochondrial membrane potential [98]. As stated, IRI also promotes the disassembly of supercomplexes, further compromising ATP production [62]. Insufficient ATP production impairs crucial repair machinery and compromises overall renal function, especially in tubular cells [7,84]. Overall, these observations highlight the need to identify mechanisms that regulate metabolic recovery post-IRI.

In response to damage incurred during IRI, mitochondrial dynamics are altered, and mechanisms that promote recovery/clearance are activated. In a typical cell, mitochondria maintain homeostasis by dividing and elongating via fission and fusion, respectively [99]. Fission is exacerbated by renal IRI, contributing to overall mitochondrial fragmentation [100,101]. This post-IRI fragmentation is specifically associated with dynamin-related peptide-1 (Drp1), a protein that is dephosphorylated at its Ser637 residue and recruited to the outer mitochondrial membrane to perform the “pinching” function required for fission [102,103,104]. Perry et al. [105] and others have reported that deletion or inhibition of Drp1 during renal IRI decreases mitochondrial fragmentation and reduces tissue injury [104,106]. When mitochondria are fragmented beyond repair or mitochondrial DNA is damaged, they are engulfed by lysosomes in a process known as mitophagy [107]. Mitophagy is a vital process in limiting mitochondrial ROS production and tissue repair [108]. Induction of mitophagy has been demonstrated to reduce renal damage due to IRI [109,110], while loss of mitophagy sensitizes cells to oxidative stress [111].

Excessively fragmented mitochondria that are not cleared via mitophagy release DAMPs, such as mitochondrial DNA or cardiolipin. These factors activate pro-inflammatory platforms, including the NLRP3 (nucleotide-binding domain, leucine-rich-repeat containing family, pyrin domain-containing 3) inflammasome [112] or the complement system [10]. Crosstalk between inflammatory signals propagates a robust sterile immune response, exacerbating tissue injury [113,114]. Specifically, complement activation has been tied to downstream inflammasome activation in monocytes/macrophages, cell types that also contribute to renal IRI [115,116].

While prior studies have identified several points of mitochondrial dysfunction during IRI, the driving mechanisms behind this dysfunction are not completely understood. For example, the ultimate source of mitochondrial ROS is not clearly defined, although prior research suggests that reduced ETC function during IRI is a major contributor to ROS [35]. Various inflammatory mediators act as another potential source of ROS that perpetuates mitochondrial dysfunction [117]. In a murine IRI study by Zhao et al., decreasing mitochondrial ROS levels attenuated renal dysfunction, mitochondrial damage, and inflammation [118]. Therefore, there is an urgent need to identify and mitigate levers of ROS production to promote renal tissue repair.

#### 3.3.2. CKD and Mitochondrial Dysfunction

While acute mitochondrial damage exacerbates renal injury, it is also critical to consider the long-term impacts of mitochondrial dysfunction in renal tissue. Direct damage to cells/cell death results in the release of DAMPs, which act as a signal to propagate inflammation within the kidney [117,118]. Excessive inflammation surrounding tissues not only results in more direct damage but is also linked to profuse extracellular matrix deposition and scarring [8]. Fibrosis leads to the permanent loss of renal function, representing the transition point from IRI to CKD. Termed a “silent catalyst” of CKD progression, mitochondrial dysfunction continues to occur in diseased kidneys as the remaining mitochondria are unable to meet the organ’s high energy demands [119,120]. Pro-inflammatory signals are continuously propagated, activating immune pathways such as the complement cascade and hampering repair. Ideal therapeutic angles for CKD will seek to mitigate both inflammatory processes and underlying mitochondrial dysfunction.

## 4. The Complement System

**Figure 5:**
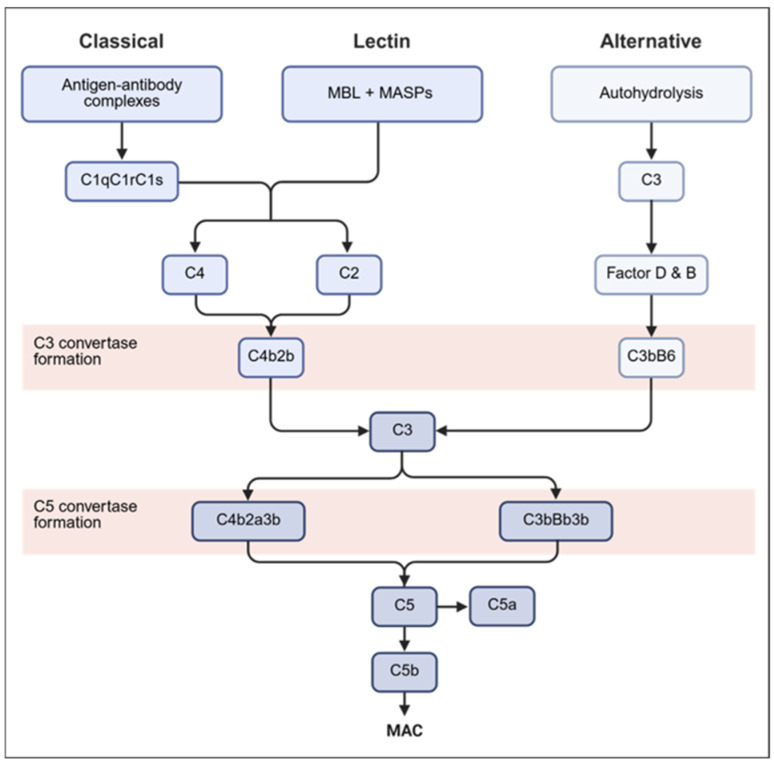
The canonical complement cascade. Created in BioRender. McGraw, M. (2026) https://BioRender.com/je0val6.

The complement system (Figure 5) is defined as a cascade of 50+ proteins that circulate within the serum and participate in innate immunity. When complement is activated in response to a pathogen or other antigen, the earlier protein components in the cascade trigger the activation of downstream protein components that culminate in a common terminal pathway and end-product [121]. The primary purpose of the end-product, termed the membrane attack complex (MAC; also commonly denoted as C5b-9), is to lyse pathogens and infected cells through the formation of pores in the cell membrane [122,123]. However, outside of infection, the complement system has more recently been recognized as a key contributor to sterile inflammation and ROS production in damaged tissues such as the kidney [124,125]. The so-called “overactivation” of complement is now linked to pro-inflammatory processes in a variety of renal diseases [126,127] with new roles for complement appearing in studies every day.

### 4.1. Production and Activation of the Complement Cascade

Recent studies have established that, in addition to “canonical” production and activation of complement proteins, multiple tissues and cell types throughout the body are capable of producing complement [14]. These findings further suggest that complement proteins play a role in physiological processes beyond strictly immune function [128,129]. Thus, the source of complement and mechanism(s) of cascade activation are crucial avenues of study when considering complement in renal pathogenesis.

#### 4.1.1. The Canonical Pathways

With limited exceptions, most soluble complement proteins that circulate in the serum are produced by hepatocytes in the liver [130]. When activated by one of three major pathways, the circulating complement components function as a proteolytic cascade that activates other inactive complement proteins, or zymogens. These pathways consist of (1) the classical pathway, (2) the lectin pathway, and (3) an alternative pathway [131]. The classical pathway is most often associated with the complement cascade’s innate immune response to infectious pathogens, fulfilling complement’s “canonical” role [132]. It is initiated when C1q, as part of a complex with complement components C1r and C1s, binds to the Fc regions of either IgG or IgM. From this initial activation, the C1s component is responsible for the cleavage of C4 and C2 into their “a” and “b” fragments, respectively. In complement nomenclature, smaller cleavage fragments are designated as “a” (i.e., C4a) while larger cleaved products are designated as “b” (i.e., C4b) [133]. From the cleaved products of C4 and C2, the C4b and C2b fragments are recruited to form an enzymatic complex, C4b2b. This complex, termed the “C3 convertase,” is responsible for cleaving C3 into C3a and C3b.

The formation of the C3 convertase is where the major canonical complement pathways differ, as each pathway forms the C3 convertase via a slightly different route. For example, in the lectin pathway, mannose-binding lectin (MBL)-associated serine proteases (MASPs) are responsible for cleaving C4 and C2 to form the components needed for the C3 convertase [134]. Conversely, in the alternative pathway [135,136], autohydrolysis of C3 occurs slowly but continuously, a process known as “tickover.” The C3b fragment spontaneously associates with Bb, a fragment of another factor in the complement cascade, forming the C3bBb alternative pathway C3 convertase [131]. Once a C3 convertase enzyme is formed, it is capable of repeatedly cleaving C3 molecules to form more C3b products. C3b can form additional C3 convertases, an important amplification step after the initial activation of the complement cascade, or it can form multimeric complexes with C4b2b or C3bBb. These multimeric complexes (C4b2bC3b or C3bBbC3b) are also known as the C5 convertase, another major enzyme of the complement cascade that marks the beginning of the terminal complement pathway by cleaving C5 into its fragments, C5a and C5b. The “terminal” pathway is the portion of the cascade that leads to the formation of the end-product of complement activation, MAC. It is facilitated by the C5b fragment, which binds to C6-C8 and multiple C9 molecules to insert pores into the cell membrane. While MAC carries out cell lysis, it is crucial to recognize that other products of the complement cascade (such as C3a and C5a) [137] have additional roles and functions.

#### 4.1.2. Intracellular Complement

In recent years, the production of complement by a variety of cell types and tissues has been recognized. Notably, both immune cells and solid organs (primarily epithelial cells) are capable of synthesizing their own intracellular complement proteins [138,139,140,141]. This production of complement proteins outside of hepatocytes, which is often implicated in autocrine or paracrine functions [142,143,144], is termed the “complosome.” Studies have suggested that local complement proteins may be involved in cell proliferation [15], metabolism [130,145], and other tissue repair mechanisms [10,14]. In the kidney, a variety of cell types readily synthesize complement proteins, including proximal tubular cells [140,146,147], glomerular epithelial cells [141], and others [148,149]. It is therefore essential to consider not only the infiltration of circulating complement, but also local complement production when studying complement-related renal pathologies.

Compared to extracellular liver-derived complement, which patrols for pathogens and promotes inflammation/pathogen destruction [124,134,150], the role(s) of intracellular complement in the kidney are not as well-defined. Renal tissue is also sensitive to complement dysregulation, although whether the source of complement is extracellular or intracellular remains largely unknown [151]. It is likely that both extracellular and intracellular complement are key contributors to renal IRI, with extracellular complement functioning as a driver of the sterile immune response [124]. If complement activation is controlled, it can facilitate clearance of cellular debris and tissue repair, although overactivation of complement is deleterious and leads to renal fibrosis post-IRI [10]. Pratt et al. reported that local C3 synthesis modulated renal transplant rejection [152], and subsequently Sheerin et al. reported that the C3^−/−^ kidneys placed in C3^+/+^ mice reduced tubular injury despite abundant circulating C3 [153]. Similarly, cytokines IL-2 and INFγ upregulate C3 mRNA expression in renal cells during IRI [154], potentially via methylation of the C3 promoter [155]. Increased local C3 synthesis could contribute to activation of the alternative pathway, generating downstream mediators such as the C3a/C5a anaphylatoxins. Intracellular C3a/C5a binding to their respective receptors has been linked to cellular homeostasis [156,157], metabolism [130,158,159], proliferation [15,160,161], and inflammasome activation [16,114]. These prior studies support an important role for intracellular complement in renal IRI pathology, even if the mechanism has not yet been defined.

### 4.2. Complement 5 and Renal Injury

While it has long been established that both complement activation and mitochondrial dysfunction are contributors to IRI and chronic disease, the mechanistic relationship between these two pathways remains incompletely understood. An early study by de Vries et al. [162] provided evidence that complement anaphylatoxins such as C5a mediate IRI through a mechanism beyond classical pro-inflammatory function. Subsequent studies also revealed complosome regulation of fundamental cellular processes, including metabolism, survival, and stress responses [10,14]. Emerging evidence indicates that the activation of certain complement receptors (C3aR and C5aR1) on the outer mitochondrial membrane promotes ROS generation [16,159,163,164], triggers mitochondria-dependent apoptosis [165,166], and drives renal fibrosis [167,168]. Together, these findings position the terminal complement cascade as a convergence point of inflammatory and metabolic injury pathways. As such, targeting complement represents a promising therapeutic strategy to simultaneously mitigate inflammation, oxidative stress, and mitochondrial dysfunction during kidney injury.

#### 4.2.1. Complement Activation in IRI

Common hallmarks of IRI include necrosis and inflammation of the renal tissue [169,170]. As discussed, DAMPs released from necrotic tissue aberrantly activate the complement cascade [171,172] primarily via the alternative pathway [11,173,174]. Because this pathway is initiated by spontaneous C3 hydrolysis and rapidly amplifies C5 activation, both C3 and C5 have emerged as major therapeutic targets in IRI and subsequent kidney disease [171,175,176].

#### 4.2.2. C5: A Clinically Appropriate Target

There are currently no effective clinical treatments available for IRI. However, due to the contributions of complement activation to IRI pathology, complement proteins are an attractive therapeutic target. C3 and C5 are often modified/targeted in IRI studies, largely due to their commonality between all 3 complement pathways and the contribution of the C3a/C5a anaphylatoxins to IRI pathology. Modulating C3 also has the downstream effect of mediating C5 activation, as C3b is a crucial component of the C5 convertase. Thus, studies that target C3 may be unable to rule out C5-mediated protective effects as a contributor to the results observed. Due to the complexity of the complement cascade and its involvement in a variety of key physiological functions, targeting the terminal cascade offers protection without interfering with the roles of upstream complement proteins. Several prior studies using animal renal IRI models have demonstrated that targeting C5 alone is sufficient to reduce tubular injury and improve renal function [12,13,168,174,177]. Similarly, the C5a anaphylatoxin exhibits greater potency than C3a and significantly contributes to renal inflammation post-IRI [12,178,179]. For these reasons, C5 may be a more appropriate target than C3 during renal IRI.

One of the first FDA-approved anti-C5 drugs, Eculizumab [180], has previously been investigated for renal injury in the context of kidney transplantation [181,182,183]. The results of such trials have been inconclusive, partially due to the poor response to Eculizumab in patients with certain C5 genetic variants [184]. This highlights the need for more nuanced anti-C5 treatment strategies to address renal injury and disease. For example, C5’s cleaved fragments—C5b and C5a—progress tissue damage and inflammation in disease states such as AKI [13,176]. Understanding the contributions of each fragment to kidney injury is critical to the future of AKI/kidney disease therapeutics.

C5b and MAC: While several studies in rodent models have demonstrated that inhibiting C5 as a whole can alleviate kidney injury [12,177,185], the role of C5b in the kidney has been predominantly studied in the context of MAC (or C5b-9). In a 2000 study, Zhou et al. indicated that tubular epithelial cells were a key target for MAC in the setting of AKI [13]. Our group corroborated this in a previous study, wherein increased MAC deposition was observed in rats exposed to injury via a kidney transplantation model [186]. Similarly, elevated MAC levels have been detected in the serum of deceased kidney donors [187], but this could not be definitively linked to kidney transplant outcomes or AKI-mediated effects. In general, the study of C5b/MAC in the context of renal injury is limited by the lack of targeted inhibitors for these proteins, and prior studies have primarily employed anti-C5 strategies [13,187] to observe MAC-related effects. In comparison, studies focusing on the C5a fragment are more common, likely due to the ability to target the anaphylatoxin receptor C5aR1.

C5a and C5aR1: Initially characterized in 1991 [188], the rhodopsin-like receptor for C5a (C5aR1) is capable of binding C5a with high affinity (K_d_ ~1 nM), thus initiating a G-protein cascade that can perpetuate a variety of cellular effects [189]. One of the most well-known effects is chemotaxis, a functional response for which the term “anaphylatoxin” was coined [150]. C5a in particular is a powerful chemoattractant for macrophages [190], neutrophils [191], B cells [192], and T cells [193], exacerbating inflammation in disease states such as AKI [175].

C5aR1 is expressed on a variety of cell types. This includes predominantly immune cells such as neutrophils and macrophages [194] but also non-immune cell types, as reviewed in Monk et al. [195] In the kidney, C5aR1 is detected on interstitial macrophages as well as in distal and proximal tubular regions [167]. When binding, C5a interacts with C5aR1 via two sites—the N-terminus of C5aR1 interacts with the core of C5a, and the C-terminus of C5a interacts with a hydrophobic binding pocket formed at the base of C5aR1’s extracellular loops [189]. This C5a-C5aR1 interaction has been linked directly to the development of fibrosis after IRI [167,168]. Furthermore, in a study by Arumugam et al., a cyclic C5aR1 antagonist attenuated renal injury in a rat model of IRI [176]. This was expanded upon in a study by de Vries et al. in the same year, which determined that C5a mediated renal injury independent from its role in chemotaxis [162]. Therefore, it is clear that the C5a-C5aR1 axis contributes to renal injury beyond its traditional pro-inflammatory role(s).

### 4.3. C5, C5aR1, and Mitochondrial Pathology

As discussed previously, inflammatory mediators such as the complement cascade (and specifically, potent complement anaphylatoxins such as C5a) contribute significantly to ROS production and mitochondrial dysfunction during AKI [117,118]. Since the discovery of C5aR1 expression on outer mitochondrial membranes [16,145], multiple studies have similarly indicated that the C5-C5aR1 axis may play a greater role in mitochondrial function. The majority of these studies have been conducted in immune cells, demonstrating that C5-C5aR1 drives ROS production [196] and eventual activation of the inflammasome [115,139] in different immune cell subtypes.

Relatively fewer studies address the role of mitochondrial C5aR1 in non-immune cells. However, emerging work has shown that in human corneal epithelial cells [165] and renal podocytes [197], activation of C5aR1 during cellular stress or injury promotes mitochondrial fission. These findings accompany earlier work by Ishii and Rohr, wherein C5a induced mitochondrial fusion in healthy retinal epithelial cells and promoted fission/fragmentation during oxidative stress [163]. Similarly, in kidney endothelial cells, treatment with high-dose C5a was sufficient to induce mitochondria-dependent apoptosis [166]. While many of the factors governing the relationship between C5-C5aR1 and mitochondria are unknown, it is possible that tissues and cell types demonstrate differing mechanisms. Furthermore, factors such as cellular injury/stress and C5a levels may affect how the C5-C5aR1 axis impacts mitochondrial dynamics. A summary of the cell lines, injury models, C5a dosage, and overall effect on mitochondrial fission/fusion from key prior studies is included in Table 1. However, despite the key role that the C5-C5aR1 axis appears to play in mitochondrial fragmentation, ROS production, and apoptosis, it has never been fully investigated in the context of mitochondrial dysfunction induced by renal injury.

In our group’s recently published work, we examined the impact of the C5-C5aR1 axis on mitochondria in proximal tubular cells and rat kidneys [158]. Briefly, we identified that C5-C5aR1 inhibition modulated the levels of ETC complexes/supercomplexes, reduced baseline complex I respiration, and significantly increased the ATP synthase regulator IF1 in mitochondria. During chemical ATP depletion, C5-C5aR1 inhibition mediated an IF1-dependent glycolytic switch, which preserved ATP levels in tubular cells [158]. While our study did not identify a specific mechanism through which C5-C5aR1 regulates the ETC or IF1, these relationships offer a new lens to examine intracellular C5’s role in renal mitochondria.

## 5. Clinical Potential

As studies have enumerated the contribution of complement proteins to various diseases in the kidney and beyond, several complement-targeted therapies have been developed (Table 2).

Although the complement system is heavily involved in a wide range of pathologies, targeting the cascade with therapeutics presents difficulties. The cascade itself is made up of many zymogens, activated fragments, regulators, and decayed products that perform innumerable roles and interact with many different cell types located throughout the body. Given that most complement therapeutics seek to target circulating complement components, avoiding system-wide effects is typically nonviable. Targeting upstream portions of the complement cascade presents similar problems to many immunosuppressive therapies: the patient becomes vulnerable to infection. However, much of the pathological activation of complement is mediated by complement proteins located in the terminal portion of the pathway—primarily, C5 and its cleaved fragments, C5a and C5b. Targeting the terminal complement pathway presents a clear advantage over other therapeutic options, as the upstream pathways and their various effector functions can be preserved. Table 3 summarizes key renal studies that have utilized anti-C5 or anti-C5aR1 therapies, including observed impacts on mitochondrial parameters. However, current clinical evidence of C5-C5aR1 mitochondrial impacts is limited, as clinical studies do not typically examine mitochondria.

## 6. Conclusions

Ample evidence now supports that mitochondrial dysfunction is key to the progression of AKI/CKD. Specifically, disruption of renal mitochondria leads to cellular damage or even death, resulting in the release of pro-inflammatory signals which stimulate immune system overactivation. Chief among the immune components involved is the complement system, a cascade of proteins with multi-faceted roles during kidney injury and disease. Aberrant complement activation not only directly damages cells and generates ROS but also stimulates further inflammation and fibrotic changes [13,174]. Recent studies reveal that intracellular complement activity is also an integral part of normal cellular metabolism [15,128,197].The terminal complement protein C5 also appears to play a role in renal mitochondria, as the C5a-C5aR1 axis promotes mitochondria-dependent apoptosis/necroptosis [165,166] and sensitizes renal cells to injury [197]. Our group recently demonstrated that C5 deficiency and C5aR1 inhibition impacts renal mitochondria, including disruption of ETC complexes/supercomplexes, impaired complex I respiratory activity, and an IF1-dependent glycolytic switch [158]. Thus, we suggest that in addition to its inflammatory role, the complement system is deeply intwined with mitochondria and hence is of broad significance in kidney disease.

### 6.1. Limitations

Despite the preclinical evidence supporting C5’s relationship with mitochondria in the kidney and other cell types, further mechanistic insight is needed before translation to the clinic can take place. Currently, there is a need to differentiate C5-mediated effects between extracellular/intracellular sources, and processes governing intracellular C5/C5aR1 expression in renal cells are not fully defined. It is also critical to address the mechanism(s) by which the C5-C5aR1 axis modulates the mitochondrial ETC and key proteins such as IF1. Clarification of these mechanisms in preclinical studies may support the need for future translational validation, as there is comparatively little clinical evidence that C5-C5aR1 interacts with mitochondria in human kidneys.

### 6.2. Future Directions

Although clinical evidence to support the C5-C5aR1-mitochondrial axis is currently lacking, the availability of FDA-approved anti-C5 and anti-C5aR1 drugs opens the door to future translational studies. Furthermore, C5-C5aR1’s well-established role as an inflammatory mediator during renal AKI/IRI implicates the axis as a worthwhile therapeutic target. Future work should compare the benefits of targeting C5 versus C5aR1, as structural variants of human C5 may complicate clinical outcomes (as with Eculizumab) [184]. Overall, despite the need for mechanistic groundwork to establish C5-C5aR1’s pathophysiological role(s) in the kidney, C5/C5aR1 pharmacotherapeutics are potentially attractive for translational studies.

## Figures and Tables

**Table 1:** Comparison of C5a Effect on Mitochondrial Dynamics.

Study	Cell Line	Injury Model	C5a Dosage	Mitochondrial Dynamics
Wang et al. 2026 [165]	2-SV40 HCECs	Hyperosmotic stress	200 nM rC5a	↑ Fission
Ye et al. 2024 [197]	Primary human podocytes [198]	Lupus nephritis	100 nM rC5a	↑ Fission
Ishii and Rohr, 2024 [163]	ARPE-19	Healthy and 0.5 mM H_2_O_2_	52 nM rC5a	↑ Fusion in healthy cells; ↑ fission post-H_2_O_2_

**Table 2:** Complement Inhibitors.

Target	Drug	Type	Approval Status/Stage	Clinical Indication	Relevant Studies
MASP-2	Narsoplimab	Antibody	Approved	Thrombotic microangiopathy (TMA)	Young et al., 2026 (TMA) [199]
Factor B	IONIS-FB-LRx	Antisense oligonucleotide	Phase 3 (IMAgINATION) [200]	IgA nephropathy (IgAN)	Phase 3 IMAgINATION (IgAN) [200]
Factor B	Iptacopan	Small molecule	Approved	Paroxysmal nocturnal hemoglobinuria (PNH); IgA nephropathy	APPLAUSE-IgAN (IgAN) [201]; APPLY-PNH (PNH) [202]
C3/C3b	Pegcetacoplan	Peptide	Approved	Paroxysmal nocturnal hemoglobinuria; C3 glomerulopathy (C3G)	PEGASUS (PNH) [203]; PRINCE (PNH) [204]; VALIANT (C3G) [205]
C5	Crovalimab	Antibody	Approved	Paroxysmal nocturnal hemoglobinuria	COMMODORE 1 & 2 (PNH) [206]; COMPOSER (PNH) [207]
C5	Eculizumab	Antibody	Approved	Paroxysmal nocturnal hemoglobinuria; aHUS	TRIUMPH (PNH) [208]; AEGIS (PNH) [209,210]
C5	Nomacopan	Small protein	Phase 2/3	Paroxysmal nocturnal hemoglobinuria	CONSENTII (PNH) [211]
C5	Ravulizumab	Antibody	Approved	Paroxysmal nocturnal hemoglobinuria; aHUS; myasthenia gravis (MG)	CHAMPION-MG (MG) [212]; ALXN1210-PNH-301/302 [213,214]
C5aR1	Avacopan	Small molecule	Approved	Severe ANCA-associated vasculitis	ADVOCATE (ANCA-associated vasculitis) [215]

**Table 3:** Anti-C5 Therapies in Renal Studies.

Study	Phase	Agent	Administration	Model/Participants	Outcomes	Metabolic Parameters (If Measured)
Adams et al. 2021 [216]	Preclinical	Tesidolumab	10 mg/kg weekly	Pig-to-Rhesus Kidney Transplant	Decreased early antibody-mediated rejection (AMR)	N/A
Ye et al. 2024 [197]	Preclinical	PMX53	1 mg/kg daily i.p.	Lupus nephritis (human and murine)	Suppressed C5a-mediated mitochondrial fission	Suppressed mitochondrial fission and improved fusion
McGraw et al. 2026 [158]	Preclinical	Avacopan	30 mg/kg i.p. bolus, 1 h prior to ischemia onset	Rodent IRI	Reduced IRI-mediated tubular damage	Altered mitochondrial complexes/supercomplexes/IF1; glycolytic switch; preserved ATP levels post-IRI
TRIUMPH [208]	Clinical (Phase III)	Eculizumab	600 mg weekly (4 wks), 900 mg biomonthly	Adults with PNH (n = 43; 18–85)	Reduced hemolysis and stabilized hemoglobin	N/A
AEGIS [209,210]	Clinical (Phase II)	Eculizumab	600 mg weekly (4 wks), 900 mg bimonthly	Adults with PNH (n = 29 adults)	Reduced hemolysis and improved renal function	N/A
Tan et al. 2019 [217]	Clinical (Case Series)	Eculizumab	1200 mg pre-dose; 900 mg weekly (4 wks)	Sensitized kidney transplant patients (n = 15; 42–55 yrs)	Reduced AMR	N/A
Siedlecki et al. 2019 [218]	Clinical (Retrospective)	Eculizumab	900 mg weekly (4 wks)	Kidney transplant patients with aHUS (n = 188; 2–75 yrs)	Improved survival and reduced recurrence	N/A
ADVOCATE (2021) [215]	Clinical (Phase III)	Avacopan	30 mg orally BID	Adults with ANCA-associated vasculitis (n = 27; 56–78 yrs)	Sustained disease remission	N/A
Schmidt et al. 2022 [219]	Clinical (Case Study)	Ravulizumab	3330 mg (per 8 wks)	Kidney transplant patient with aHUS (33 yrs woman)	Improved renal function	N/A
Locke et al. 2024 [220]	Clinical (Case Series)	Eculizumab	1200 mg pre-op	Human Xenotransplant (3 brain-dead recipients)	Prevented early TMA	N/A

## Data Availability

No new data were created or analyzed in this study. Data sharing is not applicable to this article.

## Abbreviations

The following abbreviations are used in this manuscript:

ADP	adenosine diphosphate
AKI	Acute kidney injury
ATP	adenosine triphosphate
aHUS	Atypical hemolytic uremic syndrome
CKD	Chronic kidney disease
C3G	C3 glomerulopathy
DAMPs	damage-associated molecular patterns
DRP1	Dynamin-related peptide-1
ESKD	End-stage kidney disease
ETC	Electron transfer chain
eGFR	estimated glomerular filtration rate
IF1	ATPase Inhibitory Factor 1
IgAN	IgA nephropathy
IRI	Ischemia-reperfusion injury
MAC	membrane attack complex
MBL	mannose-binding lectin
MASPs	mannose-binding lectin-associated serine proteases
MG	Myasthenia gravis
NLRP3	nucleotide-binding domain, leucine-rich-repeat containing family, pyrin domain-containing 3
O_2_^•−^	superoxide anion
ONOO^−^	peroxynitrite
OXPHOS	Oxidative phosphorylation
PNH	Paroxysmal nocturnal hemoglobinuria
PRRs	Pattern-recognition receptors
ROS	Reactive oxygen species
SCr	serum creatinine
SODs	Superoxide dismutases
TMA	Thrombotic microangiopathy
